# A Single 60 mg Dose of Denosumab Might Improve Hepatic Insulin Sensitivity in Postmenopausal Nondiabetic Severe Osteoporotic Women

**DOI:** 10.1155/2015/352858

**Published:** 2015-03-19

**Authors:** Elena Passeri, Stefano Benedini, Elena Costa, Sabrina Corbetta

**Affiliations:** ^1^Endocrinology and Diabetology Unit, IRCCS Policlinico San Donato, 20097 San Donato Milanese, Italy; ^2^Endocrinology and Diabetology Unit, Department of Biomedical Sciences for Health, University of Milan, IRCCS Policlinico San Donato, 20097 San Donato Milanese, Italy; ^3^Clinical Laboratory, IRCCS Policlinico San Donato, 20097 San Donato Milanese, Italy

## Abstract

*Background*. The RANKL/RANK/OPG signaling pathway is crucial for the regulation of osteoclast activity and bone resorption being activated in osteoporosis. The pathway has been also suggested to influence glucose metabolism as observed in chronic low inflammation. *Aim*. To test whether systemic blockage of RANKL by the monoclonal antibody denosumab influences glucose metabolism in osteoporotic women. *Study Design*. This is a prospective study on the effect of a subcutaneously injected single 60 mg dose of denosumab in 14 postmenopausal severe osteoporotic nondiabetic women evaluated at baseline and 4 and 12 weeks after their first injection by an oral glucose tolerance test. *Results*. A single 60 mg dose of denosumab efficiently inhibited serum alkaline phosphatase while it did not exert any significant variation in fasting glucose, insulin, or HOMA-IR at both 4 and 12 weeks. No changes could be detected in glucose response to the glucose load, Matsuda Index, or insulinogenic index. Nonetheless, 60 mg denosumab induced a significant reduction in the hepatic insulin resistance index at 4 weeks and in HbA1c levels at 12 weeks. *Conclusions*. A single 60 mg dose of denosumab might positively affect hepatic insulin sensitivity though it does not induce clinical evident glucose metabolic disruption in nondiabetic patients.

## 1. Introduction

Osteoporosis is characterized by reduced bone mass and disruption of bone architecture, resulting in increased risk of fragility fractures which represent the main clinical consequence of the disease. Osteoporosis is a metabolic bone disease characterized by excessive osteoclast activity. The differentiation and the activity of the osteoclasts are regulated by the RANKL/RANK/OPG (osteoprotegerin) pathway. RANKL (also known as TNFSF11) is a member of the tumor necrosis factor (TNF) superfamily and, after ligation with its cognate receptor RANK (also known as TNFRSF11a), is a potent stimulator of nuclear factor-*κ*B (NF-*κ*B), mainly expressed by the osteoclasts. The pool of circulating RANKL is largely determined by the production within the bone compartment [1], where osteocytes are the major supplier of RANKL to osteoclast precursors. Increased RANKL activity has been demonstrated in diseases characterized by excessive bone loss such as osteoporosis [2]. The pivotal role of the RANKL/RANK/OPG pathway in bone resorption has rendered it a therapeutic target for osteoporosis. A fully monoclonal human antibody raised against RANKL, named denosumab, has been developed and demonstrated to be effective in inhibiting the RANKL/RANK pathway [3]. Denosumab has entered clinical practice providing an antiresorptive drug for the treatment of postmenopausal osteoporosis.

The RANKL/RANK pathway has been involved also in the pathogenesis of insulin resistance: accumulating evidences suggest that activation of the transcription factor NF-*κ*B and the downstream inflammatory signaling pathways systematically and in the liver are key events in the etiology of hepatic insulin resistance and *β*-cell dysfunction [4–7]. The RANKL and its receptor RANK have been shown to be expressed in human liver tissue and pancreatic *β*-cells. Binding of RANKL to RANK activates NF-*κ*B signaling in hepatocytes, leading to cytokine production, Kupffer cell activation, excess storage of fat, and manifestation of insulin resistance [8].

Recently, serum soluble RANKL concentrations were found to be associated with insulin resistance assessed as homeostasis model assessment of insulin resistance (HOMA-IR) and with the number of metabolic syndrome components clustering in an individual [8]. Downregulation of RANKL in nutritional and genetic animal models of insulin resistance and type 2 diabetes mellitus revealed a marked improvement in hepatic insulin sensitivity and amelioration or even normalization of glucose concentrations and tolerance and insulin signaling [8].

Therefore, postmenopausal osteoporotic women treated with the anti-RANKL monoclonal antibody denosumab might provide a human model investigating the effect of the RANKL/RANK pathway blockage on glucose metabolism.

The aim of the present study was to investigate the effect of the systemic blockage of the RANK-RANKL signaling pathway by a single 60 mg dose of denosumab on glucose tolerance and insulin sensitivity in nondiabetic severe osteoporotic postmenopausal women.

## 2. Materials and Methods

### 2.1. Patients

Fourteen postmenopausal severe osteoporotic Italian women were consequently enrolled at the Endocrinology Unit of Policlinico San Donato. Clinical data are presented in Table 1. All patients met the criteria for reimbursement of the treatment with 60 mg denosumab every 25 weeks according the Italian “nota 79”: 9 patients were affected with at least one vertebral fracture, 1 patient was affected with a hip fracture, and 4 patients were affected with neck *T*-score < −3.0 SD and a wrist fracture and/or had age of menopause earlier than 45 years and/or parents with fragility fractures. Patients were supplemented with calcium 500 mg/die and cholecalciferol 800 UI/die.

Exclusion criteria included active smoking, alcohol abuse, overt diabetes, secondary osteoporosis, concomitant glucocorticoid treatment, kidney failure, liver or heart failure, malignancies, treatment with drugs known to reduce RANKL activity (metformin, thiazolidinediones, and angiotensin-receptor blockers), and ongoing treatment with denosumab.

All the enrolled patients gave their written informed consent and the study was approved by the local ethical committee.

### 2.2. Study Design

This was a prospective study in patients never previously treated with denosumab. Patients were clinically and biochemically evaluated at baseline and 4 and 12 weeks after the first subcutaneously administrated 60 mg dose of denosumab: anthropometric measures (body weight, height) were recorded; bone metabolism was investigated by measurement of serum calcium, albumin, phosphate, total alkaline phosphatase (ALP), PTH, and 25-hydroxyvitamin D (25OHD) on blood samples collected after an overnight fasting. serum calcium, phosphate, and albumin were measured according to routinely used laboratory kits. Serum PTH was assayed by electrochemiluminescence on an Elecsys 2010 (Roche Diagnostics, Mannheim, Germany), while serum 25OHD was assayed by a chemiluminescent assay (LIAISON test, DiaSorin Inc., Stillwater, MN, USA). Albumin-corrected calcium was calculated according the following formula: serum calcium (mg/dL) −  0.8 ∗ [4.0 – albumin (g/dL)].

### 2.3. Oral Glucose Tolerance Test (OGTT)

All the participants underwent a 75 g OGTT test after a 12-hour overnight fast. OGTT was performed according to a standardized protocol. Fasting blood samples were collected before administration of 75 g glucose solution, which was consumed within 5 min. Additional blood samples were drawn at 0, 30, 60, 90, and 120 minutes for the determination of plasma glucose and serum insulin levels. All patients were restricted from eating and drinking during the testing period. Glucose tolerance status at baseline (normal glucose tolerance (NGT): fasting plasma glucose (FPG) < 110 mg/dL and 2 h-PG < 140 mg/dL; impaired glucose tolerance (IGT): FPG < 126 mg/dL, 140 mg/dL < 2 h-PG < 200 mg/dL; type 2 diabetes: FPG ≥ 126 mg/dL, 2 h-PG ≥ 200 mg/dL) was defined according to the ADA 1997 criteria [9]. All patients were tested by OGTT at baseline and 4 and 12 weeks after the first single 60 mg dose of denosumab. Plasma glucose levels were measured using the glucose oxidase method (Roche Diagnostic Gmbh, Mannheim, Germany). Insulin levels were measured using chemiluminescence assay (ECLIA, Roche Diagnostic Gmbh, Mannheim, Germany). HbA1c was measured by a turbidimetric inhibition assay (TINIA, Roche Diagnostic Gmbh, Mannheim, Germany).

We estimated the glucose response to the oral glucose load by calculating the ΔAUC (area under the curve) of glucose using the trapezoidal integration rule. We further assessed the insulin sensitivity from the OGTT according to the commonly used surrogate marker Matsuda index [10], including plasma glucose and serum insulin taken at 0, 30, 60, 90, and 120 min during the OGTT. First-phase insulin secretion (*β*-cell function) was estimated from the OGTT by the method of the insulinogenic index, modeling the change in serum insulin divided by the change of plasma glucose from 0 to 30 min [11]. We also estimated hepatic insulin resistance index (HIRI) by AUC_G_0–30 × AUC_I_0–30, where AUC is the total area under the curve of glucose and insulin, respectively, in the interval between 0 and 30 min. AUCs were estimated using the trapezoidal integration rule and with glucose, insulin, and time expressed as mg/dL, mUI/mL, and minutes, respectively [12].

### 2.4. Statistical Analysis

Data are presented as mean ± SD. Continuous variables evaluated at baseline and at 4 and 12 weeks were compared by ANOVA. *P* levels < 0.05 were considered statistically significant. Variables with nonnormal skewed distribution (calculated ΔAUC glucose and HIRI values) were logarithmically transformed before analysis. Statistical analysis was performed by the Winstat Statistics for Microsoft Excel 2007.

## 3. Results

### 3.1. Effects of the Single 60 mg Dose of Denosumab on Bone Metabolism

All patients were tested in conditions of vitamin D sufficiency (serum 25OHD > 20 ng/mL); mean serum 25OHD levels did not vary at 4 and 12 weeks (Table 1). A single subcutaneously (sc) administered 60 mg dose of denosumab induced significant decreases in mean serum albumin-corrected calcium (9.6 ± 0.6 versus 9.1 ± 0.4 mg/dL; *P* = 0.01), phosphate (3.4 ± 0.3 versus 3.1 ± 0.5 mg/dL; *P* = 0.02), and ALP levels (81.6 ± 26.8 versus 72.2 ± 24.4 UI/L; *P* = 0.001) at 4 weeks (Table 1). Concomitantly, an increase in mean serum PTH level was detected though it was not statistically significant (89.4 ± 59.6 versus 59.1 ± 34.1 pg/mL at baseline, *P* = 0.08). At 12 weeks from the single dose subcutaneous injection, mean serum albumin-corrected calcium level was significantly reduced (9.0 ± 0.3 mg/dL; *P* = 0.01 versus baseline), while mean serum ALP level was further decreased determining an inhibition of 35% of the basal level (53.2 ± 14.1 UI/L; *P* = 0.001 versus baseline; Table 1). Mean serum phosphate and PTH levels returned to basal levels (3.2 ± 0.6 mg/dL and 74.8 ± 36.6 pg/mL, resp.).

### 3.2. Effects of the Single 60 mg Dose of Denosumab on Glucose Metabolism

Mean fasting plasma glucose and serum insulin levels were unaffected after 1 and 3 months from a single 60 mg dose of denosumab administration (Table 1). OGTT at basal evaluation diagnosed 4 patients with impaired glucose tolerance (IGT) and 1 patient with diabetes. At 4 and 12 weeks from denosumab administration, the prevalence and the diagnosis of the glucose alterations were unchanged. Insulin resistance was evaluated by HOMA-IR calculation: at baseline, HOMA-IR was higher than 2.5 in 5 of 14 patients and the administration of the single 60 mg dose of denosumab did not change the prevalence of HOMA-IR impairment in the studied group. Moreover, mean HOMA-IR values did not vary significantly at 4 and 12 weeks after denosumab administration (2.2 ± 1.7 at baseline versus 2.0 ± 1.4 at 4 weeks versus 2.7 ± 3.1 at 12 weeks, *P* = NS).

Considering the parameters derived from the OGTT, the mean AUCs of glucose levels after the oral glucose load, exploring the variations of plasma glucose in response to the glucose load, did not show significant differences between baseline and 4 and 12 weeks (Table 1). The whole body insulin resistance Matsuda index did not vary from baseline (5.3 ± 3.0) to 4 weeks (5.8 ± 3.2) and to 12 weeks after single 60 mg dose of denosumab (6.6 ± 4.7). Similarly, the insulinogenic index, exploring the defect in insulin secretion, was unaffected by the administration of a single 60 mg dose of denosumab (0.9 ± 0.4 at baseline versus 0.5 ± 0.4 at 4 weeks versus 0.8 ± 0.6 at 12 weeks; *P* = NS). Calculation of the hepatic insulin resistance index, which has been demonstrated to selectively quantitate hepatic insulin resistance in nondiabetic subjects [12], showed that a single 60 mg dose of denosumab significantly reduced the hepatic insulin resistance after 4 weeks since its administration (log HIRI: 6.6 ± 6.5 versus 6.4 ± 6.3, *P* = 0.01). The sample size, though small, was sufficient to detect a significant difference between baseline and 4-week HIRI values in the present patient series. Indeed, the reduction could not be further detected after 12 weeks (log HIRI 6.6 ± 6.7). Finally, circulating HbA1c levels showed a trend towards reduction between baseline and 4-week evaluation (37.6 ± 5.4 and 37.8 ± 4.2 mmol/mol, resp.) and 12 weeks after the injection (36.4 ± 5.6 mmol/mol; *P* = 0.04).

## 4. Discussion

Denosumab is a potent, targeted, and reversible inhibitor of bone resorption. Its clinical efficacy in increasing the bone mineral density in postmenopausal women and reducing the risk of vertebral, nonvertebral, and hip fractures is demonstrated up to 8 years of treatment [13]. Denosumab is a fully human monoclonal antibody that binds with high specificity to human RANKL, which plays an essential role in mediating bone resorption through osteoclast formation, function, and survival. Recently, the effect of RANKL has been investigated on insulin sensitivity and glucose homeostasis in animal models of insulin resistance and diabetes [8]. High serum concentrations of soluble RANKL are associated with increased bone resorption and bone loss, which can lead to osteoporosis [14]. Besides this, circulating RANKL levels emerged also as an independent risk predictor of type 2 diabetes mellitus development. Moreover, systemic or hepatic blockage of RANKL signaling in genetic and nutritional mouse models of type 2 diabetes mellitus resulted in a marked improvement of hepatic insulin sensitivity and amelioration or even normalization of plasma glucose concentrations and glucose tolerance [8].

Based on this experimental observation, we tested whether denosumab administration could affect glucose metabolism in postmenopausal severe osteoporotic women. Here, we presented the results of a prospective study investigating the effect of a single subcutaneously injected 60 mg dose of denosumab in patients never previously treated with denosumab. Patients were evaluated at 4 weeks after injection when circulating concentrations of denosumab reach their maximal levels and at 12 weeks when circulating denosumab levels showed variable decreases even up to 10-fold the maximal levels [15].

As expected, the subcutaneous administration of a single 60 mg dose of denosumab induced significant decreases in the serum ALP levels confirming the bone antiresorptive effect of denosumab. As previously reported [16], serum albumin-corrected calcium decreased though none of the patients experienced hypocalcemia, while plasma PTH levels tended to increase. A single 60 mg dose of denosumab did not have a clinically important effect on fasting glucose and insulin as well as the response of plasma glucose to the glucose load. When we looked at specific aspects of glucose metabolism by means of validated surrogated markers derived from fasting glucose and from OGTT, we could not detect any significant effect on the hepatic insulin resistance index HOMA-IR and on the whole body insulin resistance marker Matsuda index, though mean Matsuda index values at 4 and 12 weeks showed a trend to increase with respect to baseline. Similarly, 60 mg denosumab did not affect the insulin secretion as suggested by undetectable changes of the insulinogenic index values. Nonetheless, when we specifically considered hepatic insulin resistance by calculating the index HIRI validated by Abdul-Ghani et al. [12] in nondiabetic subjects, HIRI values were significantly reduced after 4 weeks. HIRI derived from plasma glucose and insulin concentrations during the OGTT correlates more strongly with the index derived from the euglycemic-hyperinsulinemic clamp compared to the HOMA-IR, likely because HIRI takes into consideration both the basal measurement of hepatic glucose production and the suppression of hepatic glucose production during the OGTT [12]. The early glucose response during OGTT provides an index of hepatic resistance to insulin with a great selectivity in detecting changes in hepatic insulin sensitivity. The detection of reduced HIRI values after 4 weeks from denosumab injection was in agreement with the reduction of the hepatic insulin resistance reported in the animal models with blockage of the RANKL signaling [8]. Indeed, the improvement of hepatic insulin sensitivity detected at 4 weeks could not be confirmed at 12 weeks: this pattern might be related to the nonlinear pharmacokinetics of denosumab whose circulating concentrations were declining after 12 weeks from the subcutaneous injection [15]. Nonetheless, it was of interest to notice that mean HbA1c levels were significantly lower at 12 weeks compared with the circulating levels measured at baseline and at 4 weeks. Therefore, the results of our short-term investigation suggest a positive though not clinically relevant effect of denosumab on hepatic resistance to insulin in nondiabetic osteoporotic women.

A previous study explored the effect of antiresorptive drugs on glucose metabolism: authors revised in a post hoc analysis data from three randomized, placebo-controlled trials in osteoporotic postmenopausal women treated with antiresorptive drugs among which there was the fracture reduction evaluation of denosumab in osteoporosis every 6 months (FREEDOM) trial to test whether antiresorptive therapies result in higher fasting glucose or greater diabetes incidence. Over a period of 3 years, no significant changes in fasting glucose and diabetes incidence were detected between the FREEDOM cohort and its control group [17]. These data were not in contrast with the present report as we similarly could not detect clinical relevant impairment of fasting glucose and insulin levels.

Admittedly, the present study provided preliminary data that warrant further investigation. The main limits are the small size of the sample and the lack of a control group treated with placebo. Indeed, such a control group may be considered not ethically advisable as all the patients enrolled suffered from severe osteoporosis complicated with frailty fractures and with a high risk of fracture that unequivocally need to be treated. It would be of interest also to examine the effect of denosumab in severe osteoporotic women with overt type 2 diabetes to test whether denosumab treatment might improve glycemic control by reducing hepatic insulin resistance. Nonetheless, our data support the link between inflammation and disrupted glucose homeostasis through the proinflammatory nuclear factor-*κ*B (NF-*κ*B) transcriptional program [18].

In conclusion, in postmenopausal severe osteoporotic women, the blockade of RANKL by a single dose 60 mg denosumab (1) efficiently inhibits the bone resorption marker, (2) does not affect glucose homeostasis at clinical level, and (3) improves hepatic insulin sensitivity in nondiabetic patients.

## Figures and Tables

**Table 1:** Clinical and biochemical features of patients treated with denosumab at baseline and at 4 and 12 weeks after denosumab injection.

Parameters	Baseline	4 weeks	12 weeks	*P*
*n*	14	14	14	
Age, years	67.1 ± 11.6			
BMI, kg/m^2^	24.8 ± 3.7	24.5 ± 4.3	24.8 ± 4.1	ns

Bone metabolic parameters				
Serum calcium^*^, mg/dL	9.6 ± 0.6	9.1 ± 0.4	9.0 ± 0.3	0.01
Serum phosphate, mg/dL	3.4 ± 0.3	3.1 ± 0.5^*^	3.2 ± 0.6	0.02^*^
Total ALP, UI/L	81.6 ± 26.8	72.7 ± 24.4	53.2 ± 14.1	0.001
PTH, pg/mL	59.1 ± 34.1	89.4 ± 59.6	74.8 ± 36.6	ns
Serum 25OHD^**^, ng/dL	26.9 ± 6.5	30.6 ± 10.5	34.6 ± 7.7	ns
L1-L4 *T*-score	−3.3 ± 1.5			
Neck *T*-score	−2.3 ± 1.0			
Femur *T*-score	−2.2 ± 1.0			

Glucose metabolic parameters				
Serum glucose, mg/dL	91.4 ± 10.1	89.6 ± 14.1	92.1 ± 15.3	ns
Serum insulin, *μ*UI/mL	9.5 ± 6.7	8.5 ± 5.5	10.5 ± 10.1	ns
HOMA-IR°	2.2 ± 1.7	2.0 ± 1.4	2.7 ± 3.1	ns
HbA1c, mmol/mol	37.6 ± 5.4	37.8 ± 4.2	36.4 ± 5.6^§^	0.04^§^
log⁡ΔAUC glucose^∧^, mg/dL∗min	2.8 ± 2.8	2.8 ± 2.7	2.7 ± 2.7	ns
Matsuda index	5.3 ± 3.0	5.8 ± 3.2	6.6 ± 4.7	ns
Insulinogenic index	0.9 ± 0.8	0.5 ± 0.4	0.8 ± 0.6	ns
log⁡ HIRI°°	6.6 ± 6.5	6.4 ± 6.3^#^	6.6 ± 6.7	0.01^#^

^*^Albumin-corrected calcium; ^**^25-hydroxyvitamin D; °homeostasis assessment model of insulin resistance; ^∧^Δ of area under the curve of glucose; °°hepatic insulin resistance index; ns: not significant.
